# SEMAT — The Next Generation of Inexpensive Marine Environmental Monitoring and Measurement Systems

**DOI:** 10.3390/s120709711

**Published:** 2012-07-18

**Authors:** Jarrod Trevathan, Ron Johnstone, Tony Chiffings, Ian Atkinson, Neil Bergmann, Wayne Read, Susan Theiss, Trina Myers, Tom Stevens

**Affiliations:** 1 School of Information and Communication Technology, Griffith University, Brisbane, QLD 4111, Australia; 2 School of Geography, Planning and Environmental Management, University of Queensland, Brisbane, QLD 4067, Australia; E-Mails: r.johnstone@uq.edu.au (R.J.); s.theiss@uq.edu.au (S.T.); 3 Environmental Research Centre, Sohar University, Sohar 311, Sultanate of Oman; E-Mail: tchiffings@soharuni.edu.om; 4 eResearch Centre, James Cook University, Townsville, QLD 4811, Australia; E-Mails: ian.atkinson@jcu.edu.au (I.A.); wayne.read@jcu.edu.au (W.R.); trina.myers@jcu.edu.au (T.M.); 5 School of Information Technology and Electrical Engineering, University of Queensland, Brisbane QLD 4067, Australia; E-Mail: n.bergmann@uq.edu.au; 6 Discipline of Information Technology, James Cook University, Townsville, QLD 4811, Australia; 7 Marine Geophysics Laboratory, James Cook University, Townsville, QLD 4811, Australia; E-Mail: tom.stevens@jcu.edu.au

**Keywords:** commodity hardware/software, power management/harvesting, underwater wireless communications, semantic technologies, data visualisation

## Abstract

There is an increasing need for environmental measurement systems to further science and thereby lead to improved policies for sustainable management. Marine environments are particularly hostile and extremely difficult for deploying sensitive measurement systems. As a consequence the need for data is greatest in marine environments, particularly in the developing economies/regions. Expense is typically the most significant limiting factor in the number of measurement systems that can be deployed, although technical complexity and the consequent high level of technical skill required for deployment and servicing runs a close second. This paper describes the *Smart Environmental Monitoring and Analysis Technologies* (SEMAT) project and the present development of the SEMAT technology. SEMAT is a “smart” wireless sensor network that uses a commodity-based approach for selecting technologies most appropriate to the scientifically driven marine research and monitoring domain/field. This approach allows for significantly cheaper environmental observation systems that cover a larger geographical area and can therefore collect more representative data. We describe SEMAT's goals, which include: (1) The ability to adapt and evolve; (2) Underwater wireless communications; (3) Short-range wireless power transmission; (4) Plug and play components; (5) Minimal deployment expertise; (6) Near real-time analysis tools; and (7) Intelligent sensors. This paper illustrates how the capacity of the system has been improved over three iterations towards realising these goals. The result is an inexpensive and flexible system that is ideal for short-term deployments in shallow coastal and other aquatic environments.

## 1. Introduction

The marine environment is under ever increasing pressures from human activity, accidents and natural disasters. Moreton Bay, located near Brisbane in Australia, is a prime example of an environment in need of monitoring where one of the largest dugong populations [1] has been adversely impacted by recent oil spills and sediment run off from the January 2011 floods [2]. Measuring the environmental health of, and prevailing conditions in such aquatic ecosystems is paramount for developing strategies/policies to ensure sustainability into the future. *Wireless Sensor Network* (WSN) technologies are emerging as one of the most versatile means to enable such measurement (as opposed to satellite or radar-based remote sensing techniques). However, there are many significant impediments that presently restrict the adoption of WSNs in marine-based applications.

Aquatic environments are extremely challenging for remote sensing systems. Such environments are only second to space in terms of unfriendliness to equipment and the logistical difficulty of deployment and maintenance—particularly in shallow and exposed systems such as open beaches and coral reefs. WSNs deployed at sea face multiple challenges such as salt water ingress, bio-fouling of equipment, damage from wave/tidal action, limited power supply, communications range constraints, and difficulties with maintenance (to name just a few!). All of these factors considerably add to the expense of maritime environmental measurement systems, which often makes them unviable as long-term environmental monitoring tools for non-industry sector applications such as environmental research and government-based monitoring and management, particularly in developing countries.

The *Smart Environmental Monitoring and Analysis Technologies* (SEMAT) project is largely driven by the need to create a low-cost intelligent sensor network system for monitoring aquatic and coastal environments, and importantly the analysis of the data resulting in information which can be used for management and planning [3,4]. SEMAT is a multi-disciplinary project drawing on expertise from academia, scientific institutions and industry. The main development philosophy is to “cherry pick” from the range of “off-the-shelf” products to make deployment of marine measurement systems as easy and inexpensive as possible for environmental scientists and others who do not always have the required instrument systems technical skills to build complex data collection networks. The reduced cost also means that large-scale systems can be constructed that have a greater spatial density and coverage, and can collect more data about phenomena of interest. SEMAT is also flexible in that it can be tailored to a specific scientific application and the hardware used is not dependent on any one vendor.

This paper details the vision and scope of the SEMAT project. We describe how the project has evolved over three iterations of the system. The marine science applications, goals and design decisions are given and the appropriate hardware/software technologies and specifications for each version. SEMAT has been involved in several field deployments that have aided in the development of the system. We present the lessons learned from these deployments and show how subsequent versions of the system were refined in response. A simple cost benefit analysis of the system is presented that gives a breakdown of all of the components involved in constructing each version of the system. The present version (*i.e.*, raw materials and hardware) costs approximately $4,000 (Australian dollars) per buoy with the sensors accounting for roughly 50% of the total cost. This analysis indicates that the price of a SEMAT system is only bounded by the number, expense and accuracy of the sensors required. At present, SEMAT is designed for short-term deployments (*i.e.*, up to five weeks). However, through gradual refinements it is expected that maximum deployment time will incrementally increase.

This paper is organised as follows: Section 2 details the specifics of the SEMAT project including its goals, motivating philosophy and the role of the personnel. Section 3 describes the initial version of the functioning SEMAT system that focused on underwater wireless communications and power harvesting techniques. Section 4 presents the second iteration of the SEMAT system that integrates commodity hardware and smart software technologies. Section 5 discusses the third version of the SEMAT system and the refinements made to the physical enclosure, enhanced power management, adherence to emerging sensor standards, semantic technologies, and providing a robust error/quality assurance alerting system. Section 6 presents a cost benefit analysis of the SEMAT project. Section 7 provides some concluding remarks and presents the future direction of SEMAT.

## 2. Smart Environmental Monitoring and Analysis Technologies

The initial SEMAT project has been funded by a $2.8 million (Australian dollars) grant under the Queensland Government National and International Research Alliances Program (NIRAP), with co-contributions by the University of Queensland (UQ) and James Cook University (JCU), and in kind support from the Torino Foundation and Milan Polytechnic University. The funds were allocated to a fractional project manager (one day a week), two senior postdoctoral research fellowships, three PhD scholarships and 1.5 technical officers/laboratory research assistants. The partner institutions independently supported three chief investigators. There was also advice and input from various academics, researchers and field practitioners. A modest consumables budget was assigned to each of the partner institutions (approx. $40,000 each per year over three years) to develop technologies and facilitate collaboration.

This section details SEMAT's goals (as proposed in the NIRAP grant application), the philosophy that drives SEMAT, and the role of the personnel that make up the core and extended SEMAT team.

### 2.1. SEMAT Goals

**Figure 1.**
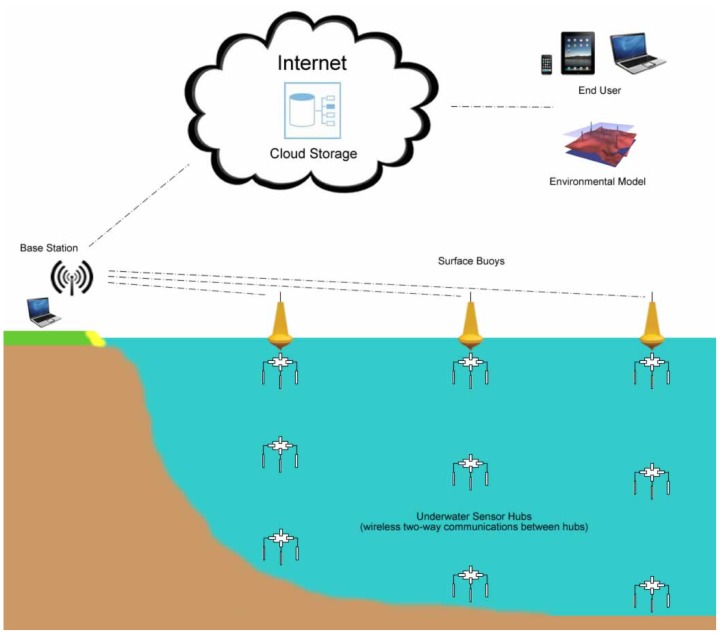
The complete SEMAT system.

The main objective for SEMAT is to determine the practicality of creating inexpensive WSNs that can be easily deployed in a marine environment with minimal expertise (see Figure 1). Here a WSN refers to a collection of sensors that wirelessly transmit data via one or more intermediate devices back to an end-user by means of a network. A sensor (or transducer) is a device that can measure some characteristic of the environment in which it is placed (e.g., temperature, light, acidity, sound, pressure, salinity, *etc.*)—a transducer takes energy from one form and converts it to another. Sensors are placed in or near the water and connect to a surface buoy that possesses computational ability. The buoy then transmits the sensed data to a base station (either located on the surface or a near-by land mass). The base station relays the data across the Internet for consumption by the end-user. The end-user is typically an individual (or group) with an interest in scientifically analysing the data and/or using it in an environmental model to gain a better understanding about a phenomenon (or series or interrelated environmental conditions).

Present marine instrument-based environmental measurement and monitoring systems are extremely expensive. Such systems have to be resilient to the elements, are often in remote places and possess limited power supply, processing power, memory capacity, and communications ability. The logistics of deploying and servicing such systems is also a severe impediment. SEMAT has tackled challenges at all of these levels to enhance the computing and maritime technologies and directly tie this in with the scientific measurement and monitoring application so that each can complement and drive the other. The overall objective is to build a system that is affordable and can be packaged as low-tech. SEMAT is based on a WSN architecture, which enables the system to be pervasive as well as “smart”. In brief the “design” goals and focus of our research has been:

1. *The ability to adapt and evolve*—SEMAT capitalises on integrating the latest trends in enhanced hardware and software technologies. SEMAT aims to use a commodity-based approach for selecting hardware and software. That is, “off-the-shelf” items that are most suitable to the application can be used during construction. For example, if the communications range increases, then all that is required is to replace the antenna with one appropriate to the desired range. Moore's Law states that every two years the power of computing roughly doubles while the size of the hardware continues to decrease [5]. SEMAT plans to capitalise on this law by letting the hardware essentially take care of itself. As such, SEMAT does not focus on the development of hardware, but rather utilises the efforts and products of others to enhance the WSN's capabilities over time.
2. *Underwater wireless communications*—Sensors wired together over any significant distance are not economically viable or practical as shallow aquatic environments are high energy, remote and often require spatially extensive data fields. Often in such environments the positioning of the cables represents a significant practical problem because the cable itself is also vulnerable to breakage or degradation over time. Likewise, acoustic modems do not work well in shallow high energy and therefore, *noisy* environments. SEMAT aims to develop underwater communications using wireless technologies.
3. *Short-range wireless power transmission*—Interconnecting cables for data communications and/or power *in-situ* is complex in a marine environment, particularly from the bottom to the surface. SEMAT has aimed to develop technologies whereby neighbouring nodes can be connected underwater and inductive methods used to transfer power and data between nodes.
4. *Plug and play*—A major problem facing the deployment of WSNs using disparate technologies (and other instrumented data collection systems), is that equipment from a variety of manufacturers must be combined that often has different communications protocols and software/hardware standards. Even the simple case of adding a new type of sensor into a system often involves reconfiguring the entire system so that the end-user can view the sensor's output. SEMAT has focussed on developing a unique software platform to allow new equipment to be added to the network such that it is instantly recognised and configured for use [6]. This is analogous to plugging in a new peripheral device for a computer such as a printer or mouse, which the operating system automatically detects and allows instant use. Making a WSN plug and play removes much of the technical overhead for calibrating, deploying and managing the network by novices.
5. *Minimal deployment expertise*—SEMAT is focused on developing a complete package. The end-user should be able to choose what sensors they require and SEMAT will auto-configure the necessary parameters. Essentially once deployed, the user should then be able use a laptop, tablet or smart phone anywhere and begin to view the sensed data with minimal post processing.
6. *Near real time analysis tools*—SEMAT must have software tools that allow data to be streamed in near real-time from sensors. Data collected is put into a format that is recognised by standards bodies (*i.e.*, Sensor Web Enablement [7]) and therefore can be imported into sophisticated data modelling, hypothesis testing and visualisation tools.
7. *Intelligent sensors*—Sensor nodes in SEMAT will have a level of intelligence allowing two-way communications between sensors and a degree of autonomy from the end-user. This way, sensor nodes can communicate with each other to alter their measurement parameters to better study the changes taking place when a sudden variation in a condition of the phenomena under study occurs. For example, if one set of sensors detects that significant rainfall is occurring, it might communicate with the salinity sensor to increase its sensing rate from hourly to fifteen minutes. There are limitless uses for such intelligence within a WSN.

Note that while the aforementioned goals are ambitious, SEMAT has not aimed to immediately deliver a fully robust system capable of being deployed long-term, in rough conditions and have sensors going down to significant depths. Instead, the SEMAT team started with short-term deployments (one to five weeks) in calm conditions at shallow depths (up to five metres). Each revision of SEMAT's development then aimed at pushing the system incrementally further towards more permanent deployments capable of withstanding extreme oceanic conditions while keeping costs down and operational effectiveness high. SEMAT is also an open design and can be used to complement existing (more expensive) measurement systems.

### 2.2. Cheaper and More Pervasive Systems—The SEMAT Philosophy

The driving philosophy behind SEMAT is that environmental measurement and monitoring systems must be increasingly inexpensive so as to become as pervasive as possible, and in this way to enable gathering of the most meaningful data streams on phenomena of interest. The data collection must also be driven by a scientific goal for its consumption, rather than “collecting data for the sake of it” [8]. This will allow sensor network systems to be more readily available to all applications and countries—especially developing countries. Such countries are particularly vulnerable to environmental damage as a result of regulatory regimes with a limited technical capacity to acquire the relevant information on impacts and changes, and a limited ability to assess such data sets using sophisticated numerical tools and strong environmental processes understanding.

In the past newly developing countries often only have access to basic information and computing technology. This is rapidly changing with the uptake of the mobile phone systems (GSM) as a global phenomenon, and the development of the Internet and more recently cloud computing in tandem with smart phones and tablets. The uptake of smart phones has meant that a leap in technology usage is now taking place from a slowly emerging localised computing capacity to a potential revolution in data acquisition and dissemination. One result is that it is now common place for instruments to be deployed in a number of regions in the world and the data sent by mobile phone networks to Europe, the US, Australia or elsewhere, post processed and the results accessed through the Web. However, despite the massive leap in our global technical ability for acquisition and dissemination of instrument-acquired data sets, there is a series of gaps in key technology and skilled capacity in newly developing countries. The resounding question this raises is, “*what is the minimum technological infrastructure required to establish an environmental data acquisition system in a developing country*”? To answer this question it is prudent to examine similar systems for developed countries.

Such a system is the Australian Integrated Marine Observing System (IMOS; http://imos.org.au/faimms.html). This project includes the deployment of remote sensing buoys at strategic locations around the Great Barrier Reef [9]. The purpose is to detect oceanographic features such as salinity, temperature, light, *etc.*, at a regional scale. However, the solution is inadequate for use in developing countries for a number of reasons. The first major shortcoming is cost. Each buoy costs an estimated $50,000−$200,000+, with deployment and maintenance also requiring significant technical infrastructure and expertise. Furthermore, this solution is not pervasive and gives only localised answers. Therefore, the effectiveness of the data for any sort of modelling is limited. As such, this sort of approach is unsuitable for a developing country.

With this example in mind, let us examine the features of cheaper alternatives. Regardless of the cost, the system must be able to deploy “enough” sensors to measure with “sufficient” accuracy to present useful information. (Note that less precise assumes sufficient accuracy.) Four million cheap sensors with limited precision are arguably of much greater value than four expensive sensors of extremely high precision. Furthermore, four million sensors will cover a larger geographical area thereby maximising the coverage for the network and environments sensed. In terms of an aquatic setting, the geographic dimensions are important. Sensor readings are not just required horizontally between locations, but also need to be at multiple points vertically between the surface and the bottom of the water body. Hence, the cost of the sensors is paramount to the amount of measurements that can be taken.

**Figure 2.**
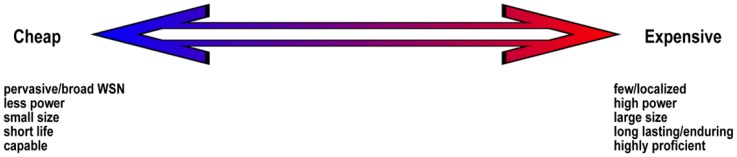
The cost spectrum for environmental monitoring systems in terms of sensor expense.

Figure 2 illustrates the cost spectrum for WSN-based environmental monitoring systems. As the design choice moves between the extremes, the features of either extreme will become more pronounced. A cheap system will allow for a pervasive network of low-cost sensors and gateways to be deployed on a potentially massive scale. Whereas expensive systems (as in the IMOS example [9]) can only result in a few sensor nodes thereby giving localised readings. Low-cost systems potentially have lower power requirements and the components are physically smaller than in an expensive infrastructure as the battery package can also be smaller. Battery size usually dictates the overall size of many small electronic commodities. However, cheap systems typically do not have the same endurance and life span as more expensive setups although bio-fouling and calibration issues still challenge both. Even so, the pace of improvement in technology means that sensors can be outdated within two/three years in any case. In general, cheap sensor nodes can be easily replaced which means their lifespan may not be a significant limiting factor. Furthermore, expensive systems that are damaged, lost or stolen are quite difficult to replace making less expensive systems more attractive. While expensive systems are highly proficient (and precise) due to their sophistication, cheap systems are usually capable albeit with less precision, but in coastal environments where spatial variability can be high as a result of strong environmental gradients this is less of an issue. This is in contrast to further offshore in oceanographic environments where gradients are small and as a result both precision and accuracy becomes particularly important.

In light of the cost structure and requirements, we define the parameters for a suitable/feasible solution for remote environmental monitoring systems in coastal environments within developing countries as follows [10]:

- The system must be cost effective, to provide the most coverage and is affordable for a developing country. (Sensors embedded in packaging can be inexpensive).
- It must use existing hardware and communication infrastructure. Even though solutions may interconnect to high technology front ends when they are available, basic, low-cost solutions that do not depend on any advanced technology are still desirable. For example, a sensor network deployment with very low-cost sensors communicating via radio or GSM mobile networks.
- The data collected must be valuable to the end-users and accessible to all interested parties. External bodies such as community groups as well as a government regulators or a United Nations agency should be able to access the interpreted findings if not also the collected data. A notable outcome in all developing countries' as a result of the now almost ubiquitous access to television, the Web and the emergence of social media such as Twitter due to the role out of GSM networks has meant that community awareness (and dissent) has increased exponentially—as most ably demonstrated by the Arab Spring. This is aiding in the establishment of sound, or in the very least accountable environmental management policies and behaviour by both government and by industry.

### 2.3. The SEMAT Team

SEMAT is a product of a multi-disciplinary effort that draws on environmental and marine scientists, computer scientists, computer systems engineers, electrical engineers, project managers and practitioners in the field. The idea is to pool knowledge and skills from experts in these areas to create a package for the end-user that abstracts complexity and makes deployment and use of the system as easy as possible.

The core SEMAT team (for the work outlined so far in this paper) consists of the following organisations:

- University of Queensland (Australia)—School of Geography and Environmental Management (GPEM, Australia; www.gpem.uq.edu.au)
- James Cook University (Australia)—eResearch Centre (eresearch.jcu.edu.au)
- University of Queensland (Australia)—School of Information Technology and Electrical Engineering (ITEE; www.itee.uq.edu.au)
- Milan Polytechnic University (Italy; www.english.polimi.it)
- The Torino Foundation (Italy)
- DHI Group (Denmark and Australia) (www.dhigroup.com)

There has also been involvement by Sohar University (Oman), Griffith University (Australia; www.griffith.edu.au), the Queensland Cyber Infrastructure Foundation (Australia, QCIF; www.qcif.edu.au) and the Australian Research Council Network on Intelligent Sensors, Sensor Networks and Information Processing (University of Melbourne; www.issensornetworkip.unimelb.edu.au)

**Figure 3.**
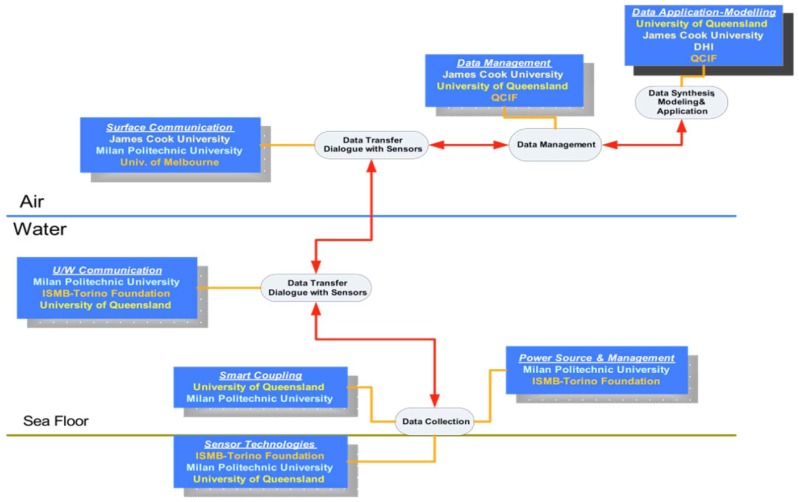
The allocation of tasks across the SEMAT project.

Figure 3 illustrates the allocation of the individual tasks and the associated distribution of technology development responsibilities for each of the SEMAT partners across the project. Tasks are divided according to work conducted in the surface/air (and back at the lab), technologies under water, and sensor devices tethered to the sea floor.

UQ (GPEM) and DHI are primarily tasked with marine science and environmental modelling. JCU is responsible for the surface hardware/software, above water communications, data storage, visualisation and semantic technologies. UQ (ITEE) is working on the underwater short-range wireless communications and inductive power transfer. The Torino Foundation and Milan Polytechnic University are developing marine sensor technologies and power harvesting techniques.

The remainder of the paper will present an overview of the work each of the partner groups made in their respective duties in realising SEMAT's goals and present an assessment of the progress we have made towards the original research objectives and what we have learnt along the way.

The development of SEMAT has been divided into three stages based on the technologies developed and the progression of time. Three different buoys (and their related technologies and deployments) have been constructed throughout the three-year period. These are referred to as the Mk1, Mk2 and Mk3 prototype buoys respectively. The following sections describe the specifics of each revision and the associated science.

## 3. SEMAT Mk1 Prototype

UQ (GPEM) (in collaboration with Milan Polytechnic University) developed the Mk1 prototype buoy as the initial attempt towards realising SEMAT's goals and was used as a proof-of-concept towards securing NIRAP funding. The Mk1 focused primarily on short-range underwater wireless communication between buoys.

**Figure 4.**
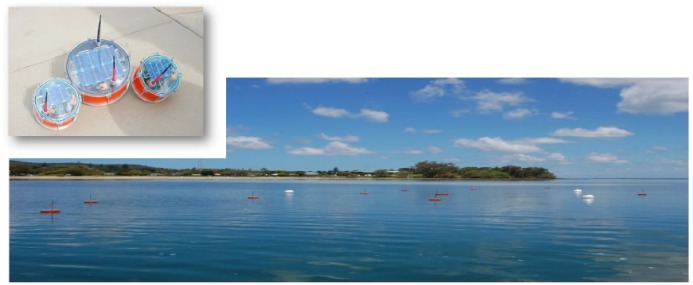
The SEMAT Mk1 prototype buoy. Examples of the gateway and surface nodes and the system deployed at Moreton Bay, Australia.

Moreton Bay in South East Queensland Australia was chosen as the site for a trial deployment of the Mk1. Moreton Bay is a National Park, contains significant Ramsa designations for wetlands and has one of the world's largest dugong populations. This site was selected due to its relevance to existing studies being undertaken by UQ (GPEM) into environmental problems in Moreton Bay and its proximity to the UQ Research Station on North Stradbroke Island, which was used as a base. Figure 4 shows the system deployed in Moreton Bay. The deployment was undertaken in June 2010 over a seven-day period.

This section describes the Mk1 system, experimentation into how underwater technologies may impact marine life, and the results from a survey into underwater power harvesting techniques.

### 3.1. Underwater Short-Range Wireless Communications

This part of the project aimed at developing a new and novel underwater antennae system. The results from laboratory and field tests showed that the Mk1 system returned data transmission rates well in excess of those achieved with commercially available acoustic modems in the same environment [3]. This is a critical aspect of the work that allows the removal of a major physical constraint on these systems—cable-based connections and the associated vulnerability and costs. The design formed the basis of the next phase of radio frequency (RF) transmission work in the project and the development of the final RF system.

The deployment consisted of 10 surface nodes, each connected to at least two subsurface sensor heads. The respective sensor heads contained sensors for water temperature and light. Figure 4 shows examples of two surface nodes with one of the surface gateways (*i.e.*, the larger buoy). The gateway acted as hub for the nodes and as the transmission-receive centre for the deployed nodes. Because of its size, the supply of power through solar and other techniques could be maximised so that it is capable of the longer distance communication (one to four km) necessary to reach either the next gateway or the base station (at the North Stradbroke Research Station).

The Mk1 used a bespoke CPU board (ATMIL CPU) with a built-in logger and 900 MHz radio. The surface nodes measured temperature and light (top and bottom), used little power, and had one-way communications (*i.e.*, from the buoys back to the gateway). It interfaced with the underwater RF and was able to deliver multiple parameter measurements at two water depths across the 10 surface nodes reliably. This represents a total of 40 data channels.

### 3.2. The Effects of Underwater Radio Frequencies on Marine Life (Elasmobranchs)

Soon after the Mk1 deployment, concerns were raised whether the underwater RF may influence the surrounding marine fauna through the alteration of natural electromagnetic fields. Elasmobranchs (sharks, skates and rays) are able to detect both electric and magnetic fields [11,12]; however, it is currently unknown how these animals may interact with submerged electrical equipment. The main objective of this study by UQ (GPEM) was to test whether wireless communication emissions from the Mk1 prototype underwater sensor hubs evoke any behavioural responses from several species of benthic elasmobranchs found in Moreton Bay, Queensland, Australia. The species studied included the grey carpet shark, *Chiloscyllium punctatum*, the estuary stingray, *Dasyatis fluviorum*, and the blue-spotted maskray, *Neotrygon kuhlii*.

Laboratory-based behavioural experiments were conducted using the underwater communication devices configured to transmit wireless radio frequency (500 Hz) signals from flat antennae at various power outputs (50–250 mW), as per the Mk1 prototype. Behavioural responses of individuals to underwater Mk1 emissions were video recorded and analysed in terms of the animal's physical response (*i.e.*, swimming or remaining motionless) and use of space, based on the calculated electric and magnetic field strengths within the experimental tank. The morphology and distribution of the electroreceptor sensory organs was also examined in all three species in order to determine potential stimuli detection capabilities.

The electric field strength emitted from the Mk1 transmissions for all power outputs was above the lower threshold limits of detection previously demonstrated for elasmobranchs [13]. Sharks and rays are typically attracted to very weak electric fields emitted by potential prey items [14–17] and repelled by strong electric fields such as those used in electronic shark deterrents. However, none of the three species showed any obvious response, either avoidance or attraction behaviours, during emission times. It is likely that the species used in this study can detect the electric field component of emissions; however, not at a level to attract or repel the animals and cause a change in their behaviour. As the magnetic field component of the emissions were much lower than magnetic fields that have elicited strong avoidance reactions in other species of elasmobranch [17,18], this may explain the lack of avoidance reactions. The results of this study are still somewhat subjective and should be interpreted with caution. Future studies should assess more elasmobranch species, both benthic and bentho-pelagic, as well as electro sensitive catfish, as interspecific variation in electrosensory detection capability may exist.

### 3.3. Power Harvesting

Milan Politechnic University commissioned a report into energy harvesting technologies for oceanic WSNs. The most promising system for underwater applications is based on piezoelectric ribbons or micro-turbines moved by the tidal current flow. Tidal turbines can be an efficient way for harvest energy from the water flow of the sea. Tidal turbines have the following main components: *Prime mover (rotor)*—extracts the energy from the water flow; *Foundation*—holds the prime mover in the flow and reacts the loads to the seabed; *Power train* (*i.e.*, gearbox and generator); and *Power take-off system* (electrical and control system). The advantage of tidal turbines is that they can be closely spaced since wake interferences are less relevant. Furthermore, tidal turbines cause less disturbance and noise for marine life as the rotor turns at very low speed. However, the marine environment is unsympathetic in terms of its impacts on materials (e.g., corrosion, marine deposits), and the watertight components increase the cost of the overall system. There are also problems of maintenance and repair. Furthermore, start-up is a significant problem as the devices are very light and only provide a low torque to the generator.

**Figure 5.**
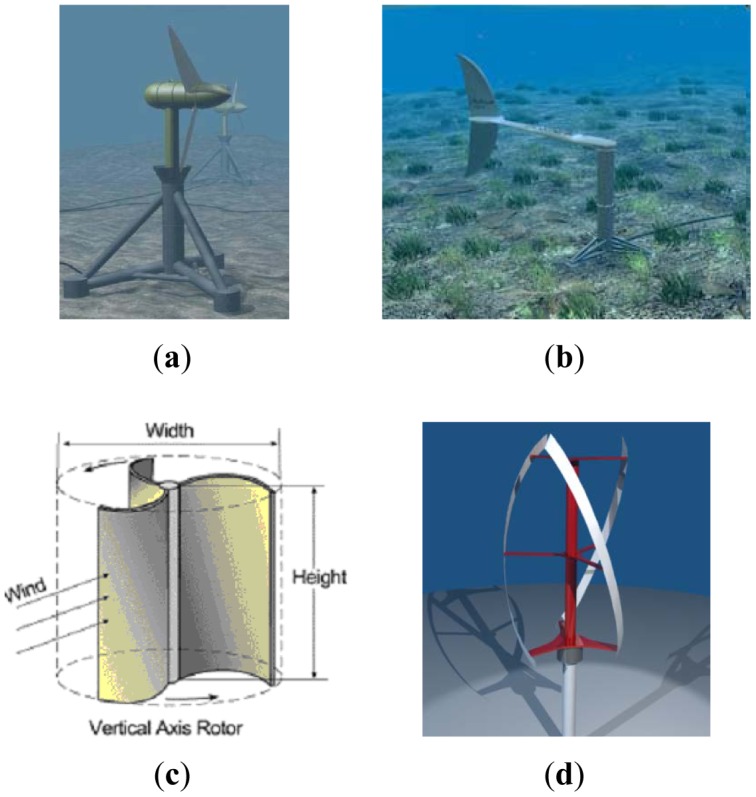
Tidal Turbines. From left to right are the (**a**) Swanturbine**^®^**, (**b**) By-ostream**^®^**, (**c**) Savonius**^®^**, and (**d**) Gorlov**^®^**.

A commercial example of a tidal turbine is the Swanturbine**^®^** (Figure 5(a)). The main problem of working with non-conventional turbines is the poor technical literature, which may be attributed to limited industrial interest for small devices, as the simulation can be very difficult due to the unpredictability of efficiency for scaled models.

Other types of energy harvesting systems reinvent traditional power systems by shifting from rotating to oscillating devices. By-ostream**^®^** is an example of this idea (Figure 5(b)). Based on the highly efficient propulsion of thunniform swimming mode species such as shark, tuna, and mackerel, it can align with the flow in any direction, and can assume a streamlined configuration to avoid excess loading in extreme conditions.

Vertical axis turbines may be more appealing for SEMAT for two reasons: (1) They have an easier adaption to periodic flows; and (2) Less problems with vibrations. At least two models potentially meet SEMAT's needs: the Savonius**^®^** and the Gorlov**^®^** turbines. Savonius**^®^** turbines (Figure 5(c)) are very easy to build and have few stresses on the primary axes. However, the torque is not smooth. To avoid this problem it is possible to use helical scoops, but they miss economic advantages. Gorlov**^®^** turbines (Figure 5(d)) are very promising, if flow remains perpendicular to the axis, regardless of the flow direction, turbine rotates always in the same direction, this makes this turbines the most efficient solution in present of flow direction variation. They can self-start with flow speeds lower than 1 m/s, and low fluctuations in torque are present in the vertical axis. Due to complex geometry, large-scale production and low power devices may not be cost effective. All are at risk in deployment due to bio fouling and entanglement from drifting alga and sea grass wrack.

### 3.4. Reflections on the Mk1 Development and Deployment

The Mk1 represented the initial attempts at bringing together the technologies that would form the greater SEMAT project and functioned as a demonstration of proof-of-concept. As such it worked but as a result of limited funds (the project was undertaken as a PhD project) and the availability of all necessary expertise, there were a number of shortcomings of the system. Some reflections on the progress for this stage of the project include:

- The technologies were principally designed for testing underwater communications.
- There were only two data streams per buoy (light and temperature). To be more useful for marine monitoring more numerous data streams would be required and from a greater variety of sensor types.
- The UQ (ITEE) and JCU teams had not yet commenced work on the project therefore there was limited progress made towards realising SEMAT's other goals.
- The system used a bespoke hardware design and therefore it was difficult to extend upon it without specialist knowledge. Due to the closed nature of the hardware developed, a completely new direction was undertaken for the Mk2 design.

## 4. SEMAT Mk2 Prototype

**Figure 6.**
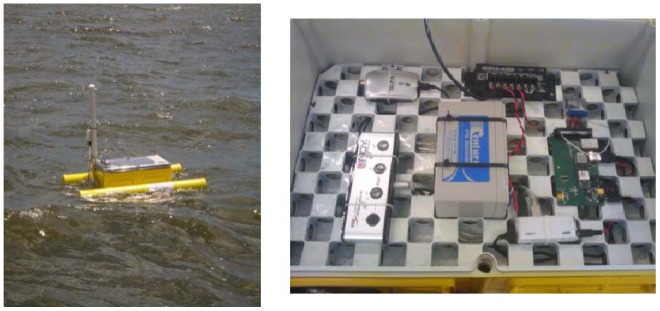
The SEMAT Mk2 prototype buoy. The illustration on the left shows the Mk2 *in-situ* at Deception Bay, the illustration on the right presents the Mk2's internal components.

The SEMAT Mk2 prototype was developed by UQ (GPEM) and JCU to create an open standard system using commodity hardware and software (consistent with Moore's Law [5]), which could integrate non-vendor specific sensor technologies (*i.e.*, making the WSN plug and play). The core component of this part of the project focused on using an inexpensive surface buoy (see Figure 6). The Mk2 buoy was designed for deployments in calm conditions over a short duration (two–three weeks).

The Mk2 deployment was undertaken in Deception Bay, Queensland Australia, for three and a half weeks during February 2011 (shortly after the 2011 Brisbane floods [2]). This is an area well known to UQ researchers as a study site for a hazardous algal bloom by a cyanophte, *Lyngbya majuscules*. Lyngbya is a particularly virulent form of cyanopyte that blooms in summer within the northern end of Deception Bay in association with seagrass beds. The alga absorbs nutrients from the sea floor sediments and surrounding waters and as a result of its prolific growth kills the sea grass. After the algae finish growing, it is broken up by storm events and typically washes up on the beach/shore line. It leaves scaring along the seabed where it was present and it usually prevents future sea grass from growing. While it is uncertain what causes the algae to bloom, it is suspected that it forms mainly in warm conditions, shallow and calm water, and human activity (possibly in the form of nutrient run off) may affect its life cycle. The initial SEMAT deployment was undertaken here as the area is relatively well protected and as a case study the data collected may be of relevance to understanding the influences contributing to the growth and spread of Lyngbya. Ideally the outcome from this was to suggest an environmental management plan to control its spread in aquatic environments.

**Figure 7.**
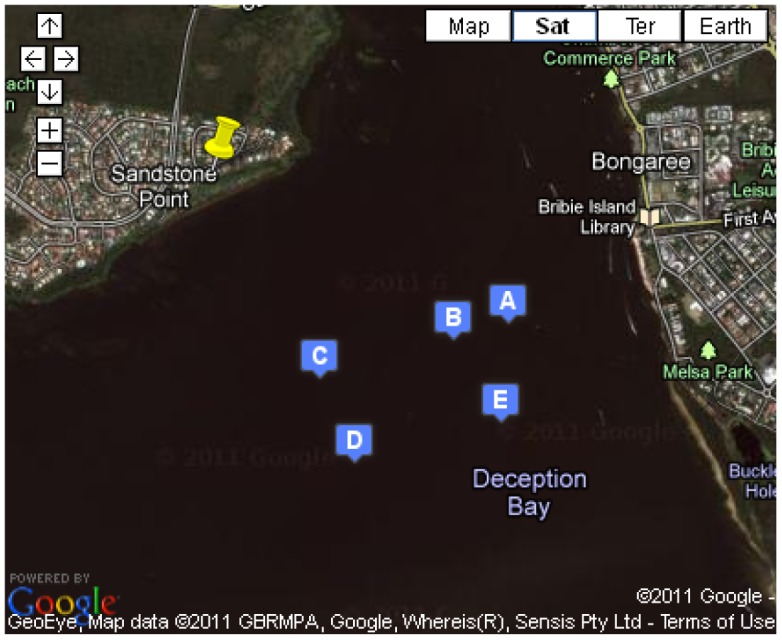
Locations of the SEMAT Mk2 buoys within Deception Bay. Each buoy was labelled A, B, C, D and E respectively. All buoys were identical except for Node A, which contained an above water light sensor. The base station was located on Sandstone Point (indicated by the yellow pin). The buoy ranges from the base station were as follows: Node A—1.4 km, Node B—1.3 km, Node C—1.0 km, Node D—1.4 km and Node E—1.7 km.

UQ and DHI Group selected Deception Bay as the site for testing the Mk2 system for the following reasons: (1) Proximity to Brisbane; (2) Prevalence of algal blooms; (3) Subtropical environment; (4) Shallow water (three metres at its tidal peak); (5) Calm conditions (it is sheltered from the ocean); and (6) Limited influence from human activity. Figure 7 shows the locations where measurements were taken within the bay.

**Figure 8.**
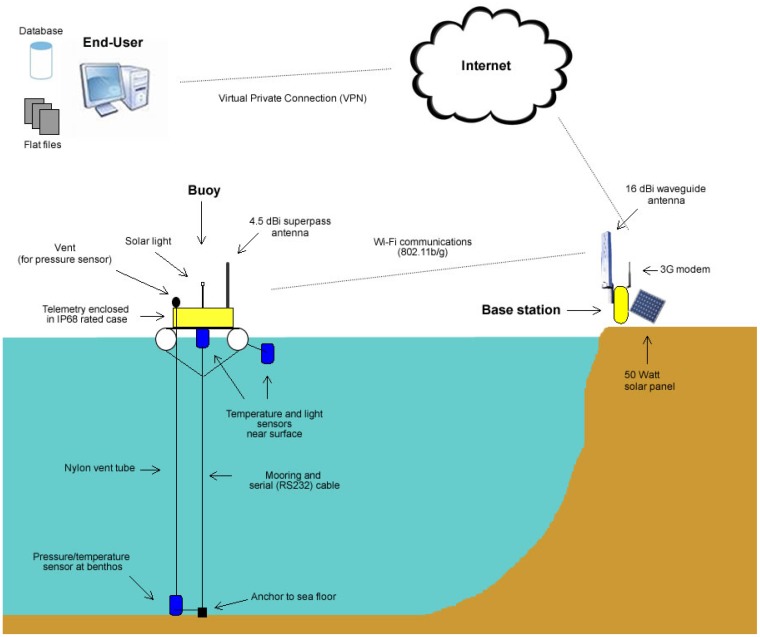
The conceptual setup for the Deception Bay deployment.

Figure 8 illustrates how the Mk2 buoy was configured (from a high-level perspective). Each buoy is tethered to the sea floor via a cable. The buoy is constructed from an IP68 rated box (dust proof and ingress protection for immersion beyond one metre) fastened to two foam filled PVC pipes via a metal bracing. The following subsections describe the development of the technologies related to the Mk2 buoy.

### 4.1. Hardware

#### Sensor nodes

The computation and communication subsystem used in the Mk2 is comprised of a Gumstix**^®^** Overo Air COM (www.gumstix.com), a Chestnut43 expansion board, and a Universal Serial Bus (USB) Wi-Fi module. A Gumstix**^®^** Overo Air COM is a 600 MHz ARM Cortex-A8 CPU with 256 MB of RAM, Bluetooth and low-powered Wi-Fi (802.11b/g). A Chestnut43 expansion board provides interfaces for Ethernet, USB A(Host)/mini-AB/mini-B, Audio, and a 40-pin header providing access to the Overo's I2C and SPI (Serial Peripheral Interface) buses, and six ADCs (Analog-to-Digital Convertor). The on-board Wi-Fi chipset was not going to be powerful enough with the maximum link distance growing from 500 m to 1.7 km, which became apparent during the evolution of the geographical configuration requirements. Fortunately, the commodity nature of our design allowed us to find a suitably powerful substitute and simply plug it into the Overo's USB port; we ended up using an Alfa AWUS036NH. However, this impacted on the power budget. A Serial to USB convertor port was also required as the Odyssey**^®^** logger/sensor only supports serial communication. A sensor node could support up to four attached sensors via a serial interface.

#### Base station

**Figure 9.**
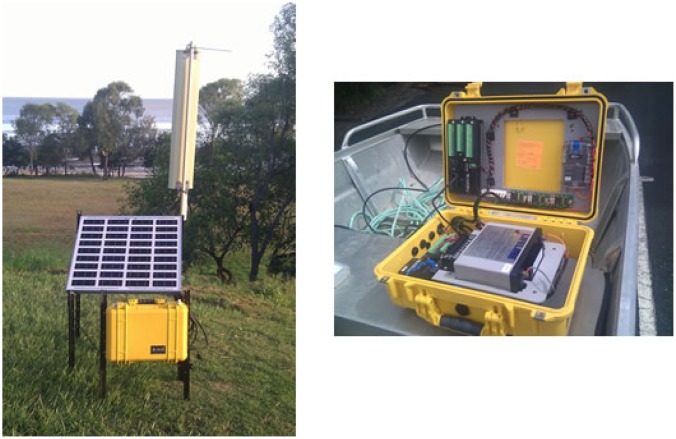
The SEMAT Mk2 base station on Sandstone Point at Deception Bay.

The base station, loaned to SEMAT by the Australian Institute of Marine Science (AIMS), was located on Sandstone Point on the Deception Bay mainland. Figure 9 shows the base station components. The base station consisted of a 16 dBi waveguide antenna and powered by a 12 volt sealed lead acid battery charged by a 50 watt solar panel. The electronics were enclosed in an IP68 rated pelican case and included a 3G modem.

### 4.2. Power Management

**Figure 10.**
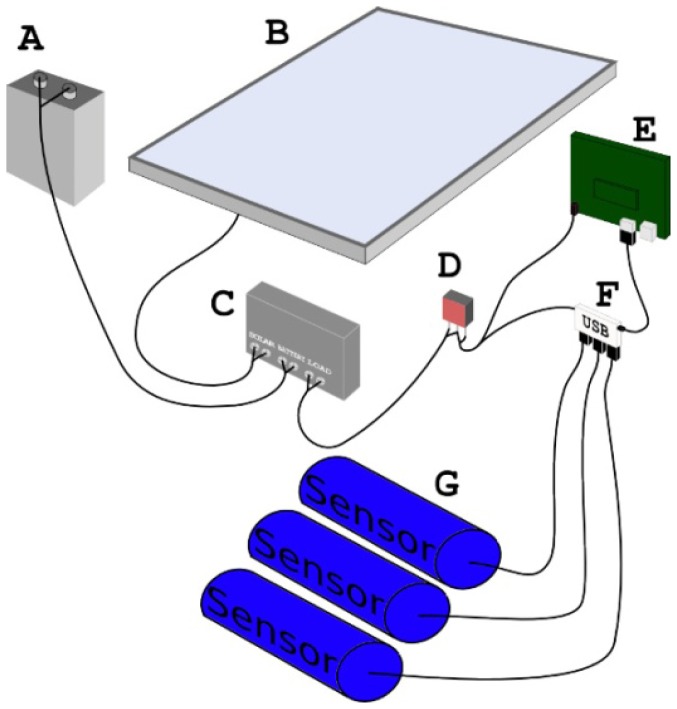
The power system for the SEMAT Mk2 prototype.

**Figure 11.**
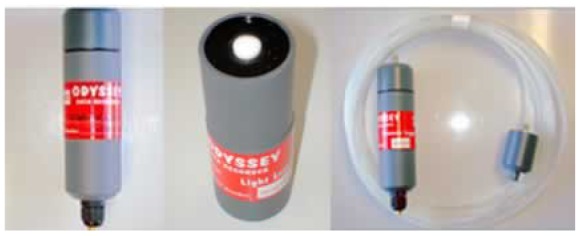
The Odyssey**^®^** data loggers used for the SEMAT Mk2 buoy. From left to right: temperature, photo irradiance (light), and combined pressure/temperature.

Figure 10 gives an overview of the power system for each buoy. The Mk2 power system consisted of elements A, B, C and D from Figure 11:

- A 12 volt 7 amp hour sealed lead acid battery;
- A 10 watt solar panel;
- A solar regulator—a Morning Star**^®^** SunSaver 6L Solar Controller. The solar regulator manages the power flow between the solar panel, the battery and the power for the rest of the system; and
- A switch mode regulator—a TRACO**^®^** TSR1-2450 DCDC 1A step down switching regulator powers the Overo**^®^** and its various components.

Components E, F and G refer to the Gumstix**^®^**, USB hub and sensors respectively. The power supply for the sensors and the maritime solar light are independent of main power supply. The antenna is not shown in the diagram.

#### Duty Cycling

This is a technique used to reduce power consumption by powering down (or hibernating) a buoy's electronics in between sensing activities. The software on the buoys has the functionality to start and stop the Odyssey**^®^** loggers on command. When starting a logger, it configured it to take an independent reading at 15-minute intervals. The station then powered down until it is instructed to turn back on, or if a predetermined cycling schedule was implemented.

In terms of the Mk2 a cycle refers to powering up, stopping all loggers, downloading their data to the Gumstix**^®^**, communicating it back to the end-user via the base station, restarting all the loggers, and then powering down again. A buoy powered up at 10 am, transmitted its data, and then powered down. It did this every two hours through to 4 pm. It stayed powered down all night until 10 am the next morning (except for a maintenance cycle at midnight). The goal was to conserve battery life by only using the system during the day when the solar panels could compensate for the power consumption. Note that was is a staggered schedule so that each sensor station gains exclusive access to the base station for the period it is active. That is, the first station cycled on from 10:00 am to 10:15 am, the second from 10:15 to 10:30, *etc.*

### 4.3. Above Water Communications

Each sensor was physically connected to the Gumstix**^®^** via serial RS-232 cables. As the Gumstix**^®^** only supported USB connections, a serial to USB port was required. Each serial cable (three in total for a sensor station) connected directly to the port. The port converted the signals to USB, which then connects to the Gumstix**^®^**. Wireless (WiFi 802.11b/g) communications were used to relay the information back to the communications gateway located on the floating platform. In the best-case scenario this had a throughput of 150 Mbit/s using a 2.4 GHz band. A 3G modem on the base station relayed this back via the Internet to an end-user. An end-user could establish a virtual private network into the system when a buoy was powered up.

### 4.4. Sensors

The *Lyngbya* study being undertaken by UQ (GPEM) and the DHI Group required several types of characteristics to be measured. The initial deployment aimed to collect the following data: *dissolved oxygen*, *light*, *pressure*, *salinity*, *temperature* and *turbidity*. The Mk2 component was only concerned with light (photosynthetic irradiance), temperature and pressure. However, there were additional measurement and logging devices placed throughout the study site (such as nephelometers) that were not part of the WSN.

Dataflow Systems is a New Zealand-based company that supplies a range of terrestrial and aquatic logging devices. These are referred to as Odyssey**^®^** loggers (www.odysseydatarecording.com; see Figure 11). A logger is designed to be a standalone unit complete with its' own casing, independent power supply and on board memory. A user initially sets up the logger by connecting it to a computer via a serial/USB cable and running Odyssey's**^®^** proprietary software. Once the logger has been started, the cable is disconnected from the device, the cap screwed on and then the device is deployed. At the end of the deployment, the device is retrieved, the cable attached, and then the data is downloaded. Depending on the sensor type, the recording interval can be set between ten seconds and eight hours. Theoretically, Odyssey**^®^** loggers can be deployed for up to 18–24 months depending on the recording interval. Each Odyssey**^®^** logger contains two 3.6 V lithium cells—7.2 V in total). Loggers are independent of the buoy's main power supply. Battery life depends upon how frequently the user logs data. For an hourly logging interval under normal operating conditions, it is estimated that batteries should last for more than 18 months.

The loggers were required to be permanently attached to a computer and able to be started and stopped via the buoy's computer during deployment (without having to be retrieved). The data were downloaded and transmitted back to the end-user. However, Odyssey**^®^** loggers are not designed for this functionality so we drilled into the existing cap of the logger and fixed a gland with an O-ring seal that tightened around the cable to keep water out. The Odyssey**^®^** protocol was reverse-engineered and a software driver created for use with SAL.

### 4.5. Software—Sensor Abstraction Layer (SAL)

Lack of standards makes it difficult to integrate heterogeneous sensors in a single WSN. Therefore it is important to have a software layer, which is able to manage all types of sensors. Such software (or middleware) is often implemented specifically for a specific sensor network making it hardware dependent. In such systems changes to the network, such as adding a new sensor, leads to the manipulation of the middleware code. To circumvent this problem a unique software solution (SAL) was implemented for SEMAT by JCU.

**Figure 12.**
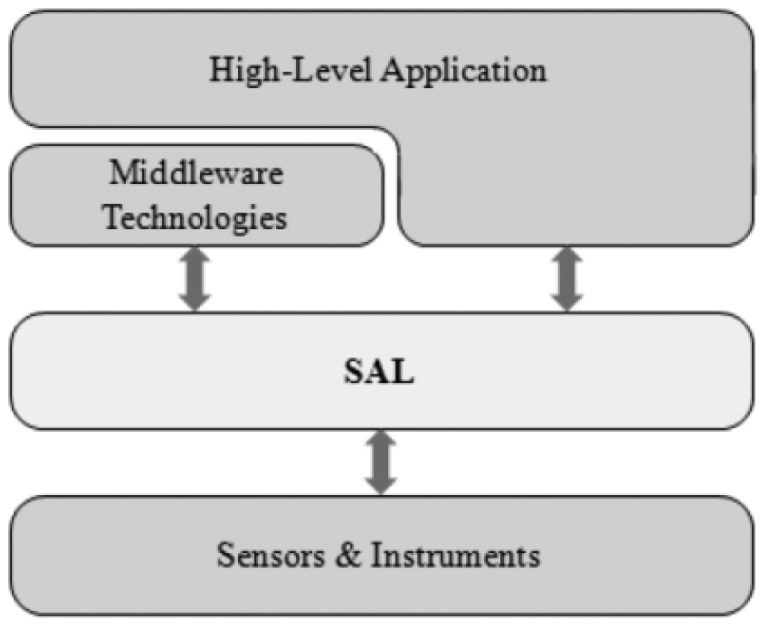
The SAL software model. SAL sits between the lower middleware and the hardware for the sensors and instruments. It facilitates a transparent interface for communication between the high-level application programs and the sensor hardware.

SAL is a middleware integration platform that provides a plug-in-based model where support for new types of sensors can be loaded to the running system. The system automatically detects and configures new sensors, if permitted by the hardware and operating system (OS). SAL provides a unified interface to all sensors by abstracting the sensor specific features, which simplifies access to a sensor network and the management/control of the sensors [6]. SAL can be seen as a low-level software layer as it bridges a network of sensors with high-level applications or further middleware technologies (see Figure 12).

SAL consists of two components, the SAL client and the SAL agent. The SAL client represents an interface for SAL to either the user via a user interface or to other applications. It implements the SAL agent Application Programming Interface (API) in order to provide the SAL agent's functionality. The API is grouped into the following categories:

- *Sensor Management*—Methods to manage the pool of sensors (enumerating, adding and removing sensors).
- *Sensor Control*—Methods to report on a sensor's capabilities and control data streaming.
- *Platform Configuration*—Methods to adjust the platform, e.g., add support for a new sensor type.

Each category uses a different mark-up language. The Sensor Management methods use a form of SensorML [7], which describes a sensor's configuration. The methods in the category Sensor Control use CommandML. The CommandML documents contain a list of commands that are supported by a sensor. The last category, Platform Configuration, uses Platform Capabilities and Configuration Mark-up Language (PCML). PCML documents contain information on the platform configuration in order to support a certain type of sensor technology. In terms of the Mk2 buoy, SAL was used to operate the Odyssey loggers.

SAL was also extended to provide an interface for the Ring Buffered Network Bus Data Turbine [19,20]. The Data Turbine is a real-time streaming data engine to stream data from experiments, labs, web cams and Java enabled mobile phones. In this instance the Data Turbine was used for streaming data from a WSN. The Data Turbine provides the end-user with a powerful visualisation tool and the ability to fast forward and rewind data.

### 4.6. Data Management and Storage

**Figure 13.**
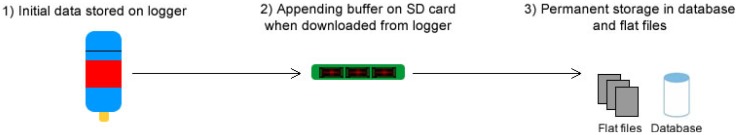
Overview of the Mk2 data backup and storage hierarchy.

Figure 13 illustrates the three main tiers to storage and back up of the data: (1) The logger (1st tier—transient); (2) An SD card attached to the Gumstix**^®^** (2nd tier—semi-permanent); and (3) The database and flat files (3rd tier—permanent)

Initially data is stored in the internal memory of the logger. Odyssey**^®^** loggers contain 64 KB of memory (65,528 bytes). The temperature and light sensors for this deployment store 2 bytes per reading. The temperature/pressure sensor records 4 bytes per reading (*i.e.*, 2 bytes for temperature and 2 bytes for pressure). The amount of memory is capable of storing 32,764 records. A scan interval of 15 min has 96 recordings each day. The total number of days is 341 days before the memory capacity will be used up. When the memory is full, the logger shuts down. Note that if the logger malfunctions before it is uploaded, then the data for that interval will be lost.

When the logger is shut down, the data is transferred to an SD card on the Gumstix**^®^**. Once the logger is started up again, its memory is cleared. The SD card contains an appending buffer. Every time data is downloaded from a logger, a copy is retained on the SD card (until the deployment finishes). Assuming a duty cycle of two hours, 64 bytes of sensor readings plus overhead will be transmitted per sensor station. (8 bytes per reading × 8 readings over two hours (15-minute intervals).)

The final tier in the memory hierarchy is permanent storage in a database or flat files. The database was implemented in MySQL and can be queried to retrieve specific data regarding the sensor readings. The flat files are straight text files where the sensed data is appended to existing records. The files can be imported into a spread sheet or used as input to other external programs.

### 4.7. Data Visualisation and Presentation

**Figure 14.**
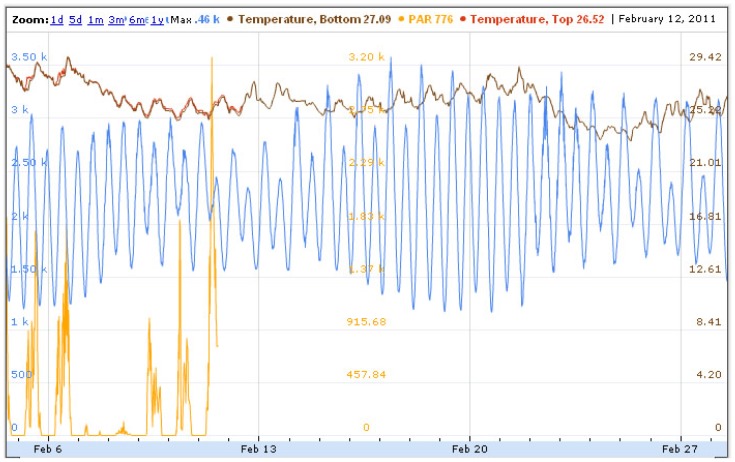
Data collected from one of the SEMAT Mk2 buoys at Deception Bay.

JCU was tasked with developing the SEMAT user interface. As data was gathered from the buoys (dependent on the duty cycle), it was calibrated and then pushed into Google Fusion Tables. To view the data for any particular node, the user clicked on the node in the Google map and a graph is displayed. Figure 14 illustrates sample data collected from a buoy at Deception Bay. Data presented includes two temperature sensors (top and bottom), light (PAR), and pressure. The graph shows that the light and surface temperature loggers failed roughly one week into the deployment (the reasons will be explained in Section 4.9).

### 4.8. Towards Underwater Wireless Communications

**Figure 15.**
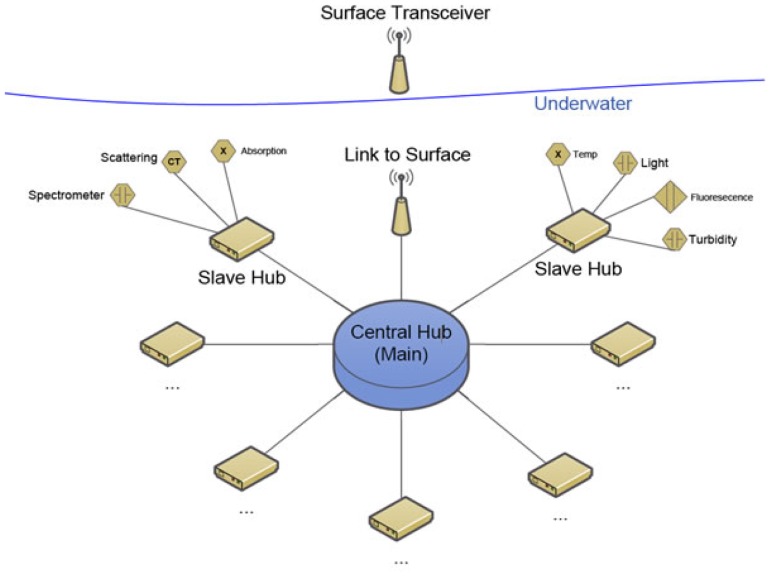
The proposed architecture for SEMAT underwater wireless sensors.

Engineers in the School of ITEE at University of Queensland developed a prototype for an underwater sensor network that can communicate with the Mk2 surface buoy. The aim was to produce a cost-effective solution utilising pre-existing technologies, which are rapidly scalable and consume minimal power; whilst providing a simple end-user experience (see Figure 15). Using existing low-cost hardware and software components assisted in these goals—the first prototype was built using low-cost Arduino controller boards, an open-source sensor node operating system (Tiny OS) customised to Arduino, and CAN-bus communications protocols [21].

**Figure 16.**
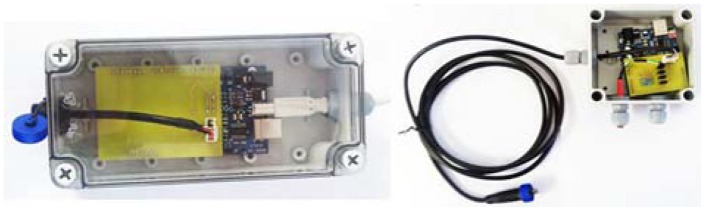
The prototype underwater sensor hubs.

There are two main components for the underwater network: the central hub and the slave hubs (see Figure 16). The slave hub provides a uniform communications interface between different types and brands of sensors and the central hub. The central hub aggregates data from the slave hubs, schedules the operation of the slave hubs including powering them down when not in use, and handles the communication and sensor interface to the surface buoy. This modular sensor architecture has been demonstrated in the lab, but robust physical enclosure and connector design would need to be undertaken before field deployment. Note that while work on this prototype was undertaken in conjunction with the Mk2's development, it did not form part of the Deception Bay deployment.

### 4.9. Reflections on the Mk2 Development and Deployment

Ultimately the scientific application of the Deception Bay deployment was a success. The data gathered enabled the hydrodynamic model to predict algal blooms to within 20 m of its actual observed locations [22]. However, there were some technical shortcomings of the Mk2 system that are outlined below.

- *86% of the loggers failed throughout the deployment's lifespan (approx. three and a half weeks)*. The main reason was that the cables used were not designed for underwater use. Some broke with the wave/tidal action. Others suffered damage to the insulation allowing water to leak internally down the cable into the logger. In some instances, loggers had become unplugged due to pulling on the cable that exceeded the gland's ability to hold it firmly in place.
- *The design of the buoys disallowed ‘hot swapping’ equipment in the field such as sensor cables, antennas and the components located within the enclosure*. From the outset, Node A had a damaged antenna cable and could not be fixed in the field. This also prevented the replacement of damaged sensors once deployed.
- *The mooring system was not particularly robust*. During the deployment, Deception Bay experienced abnormal wave action (up to three metre waves). This resulted in Node A breaking its mooring and washing up on the ocean facing side of Bribie Island. Node C was struck by a boat and was also dislodged from its mooring.
- *Not all of the equipment could be completely suspended (powered down) in between duty cycles*. This placed a constant drain on the power budget and limited the ability of some buoys to regularly establish contact with the base station—particularly on overcast days where the solar panel was unable to sufficiently charge the battery.
- *Bio fouling of equipment affected measurement accuracy*. This was most detrimental to the light sensors. Odyssey^®^ does not provide anti-fouling mechanisms for their loggers, such as wiper blades to disturb settled sediments.
- *There was insufficient data quality assurance and error alerting*. Some errors may have been due to hardware or software faults. However, there was no way to ascertain whether these had occurred and what effects they had on the system during the deployment.
- *Most of the electronics in the base station were redundant*. As AIMS loaned the base station to SEMAT, it was not tailored for SEMAT's modest needs. Therefore, the decision was made to design a less expensive, streamlined base station for the Mk3 that was consistent with SEMAT's development philosophy.
- *The buoy antenna height from the sea surface was too low*. This had a detrimental effect on communications—particularly as conditions got rough and wave action caused transmission impediments.

## 5. SEMAT Mk3 Prototype

**Figure 17.**
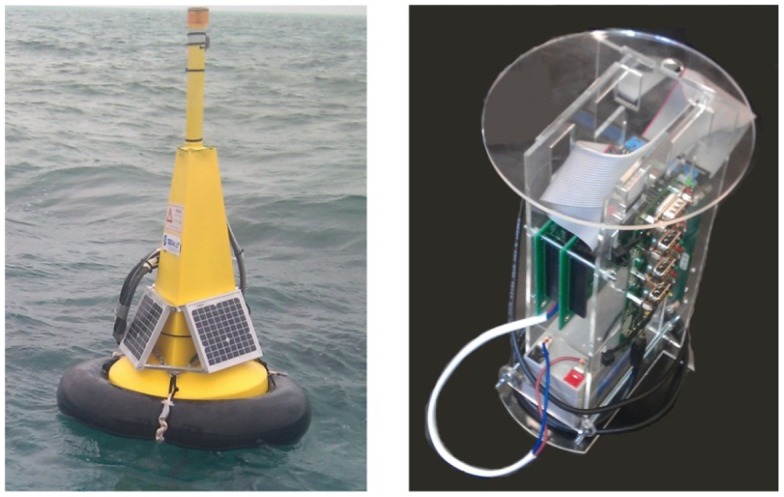
The SEMAT Mk3 prototype buoy deployed at Heron Island (**left**) and the internal electronics (**right**).

The goal for the SEMAT Mk3 prototype was to reflect upon the shortcomings of the Mk2 system and where possible ensure that the same problems either did not occur, the system could recover from detrimental circumstances, or at the very least alert the user to an issue and its potential cause. Figure 17 illustrates the Mk3 buoy (developed by UQ (GPEM) and JCU).

Heron Island near Gladstone in Queensland Australia was chosen as the deployment site for the Mk3 system. Heron Island is surrounded by a unique reef crest with an internal lagoon that fills and drains with the changes in tide. UQ has a permanent research station on the island and the area is one of the most intensely studied marine parks in Australia. AIMS have several permanent buoys positioned around the lagoon as part of the Queensland IMOS network. The deployment took place in January 2012.

**Figure 18.**
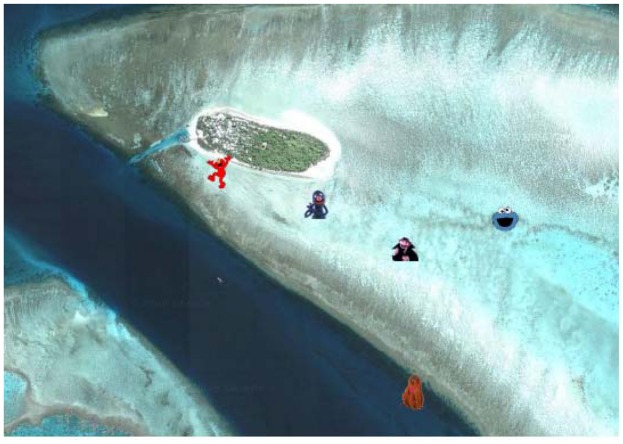
Locations of the SEMAT Mk3 buoys around Heron Island. The buoys are labeled Elmo, Grover, Count, Snuffy and Cookie respectively (from left to right). All buoys are identical. The base station is located on Heron Island.

The goals for the Heron Island deployment were to deploy five buoys for a period of five weeks (the longest period so far). Additional loggers were added to show that the design could be scaled up. The Mk3 was to bring together as much of the SEMAT technology as possible that had been developed over the prior two years within the period of the NIRAP funding. The Mk3 would integrate with the existing AIMS system at the user interface level. Finally, the Mk3 deployment was to show case the way forward for how such a system might be deployable in a developing country. Figure 18 shows the locations that the buoys were positioned around Heron Island. The deployment team consensus was that the buoys were named after Sesame Street**^®^** characters to aid in identification. Locations were selected based on environmental characteristics—such as the edge of a reef crest, shallow water, biological phenomenon, *etc.*

**Figure 19.**
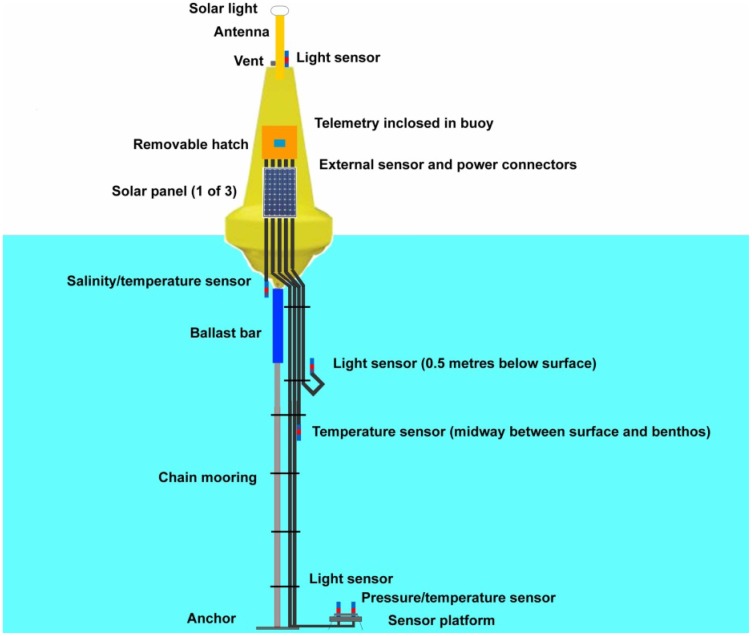
The conceptual setup for the Heron Island deployment.

Figure 19 presents the framework for the Heron Island deployment. Each Mk3 buoy contains six sensors/loggers with a total of eight data streams. The buoy was modified to include a removable hatch. The electronics are placed within the buoy. External connectors where used to allow the sensors and the solar panels to be attached/detached without having to remove the hatch (*i.e.*, the components became “hot swappable” in the field). A removable solar panel shroud was placed around the lower section of the buoy. There was a mounting platform for the antenna, detachable solar light, an above water light logger, and pressure vent (for the pressure/temperature sensor).

The most fundamental decision for the Mk3 was to use a different buoy. The Mk2 buoy design was not particularly robust (in rough sea conditions) and the antenna was too low to the sea surface, which caused issues with establishing communications. Furthermore, the design was not a maritime standard and it was feared that there could be legal ramifications if a general water user (e.g., boat, Jet Ski, swimmer, *etc.*) was inadvertently injured or suffered damage from striking the buoy. Therefore an “off-the-shelf” solution was sought.

**Figure 20.**
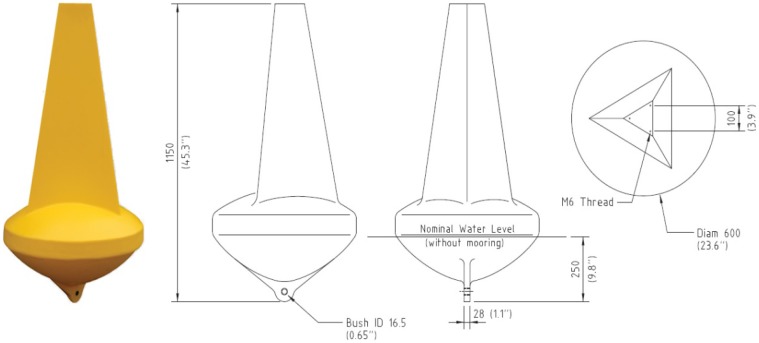
The Sealite^®^ SLB600 buoy used for the SEMAT Mk3 prototype.

The Mk3 used a Sealite SLB600**^®^** buoy (see Figure 20). These buoys are supplied by the Australian distributor Sealite**^®^** (www.sealite.com.au) and are commonly used as channel markers. As such, the SLB600**^®^** conforms to all current maritime standards. The SLB600**^®^** stands at approximately one metre above the water line, which provides more height for the antenna. The cost of a SLB600**^®^** is $195—almost 50% less expensive that the enclosure components of the Mk2 buoy. The SLB600**^®^** is comprised of UV-stabilized virgin polyethylene and is filled with closed-cell polyurethane foam. The buoy walls are 7 mm thick and it contains a stainless steel mooring bush.

### 5.1. Hardware and Power Management

The Mk3 used a newer version of the Gumstix**^®^** Overo Air COM that was smaller and doubled the amount of RAM to 512 MB. The system also changed from a Chestnut**^®^** to a Summit**^®^** board. The Summit**^®^** board used less power and contained fewer redundant features. An additional serial port hub was added that consumed less power. The new hub allowed the Mk3 to attach up to eight Odyssey**^®^** loggers (note that this can be scaled up depending on the number of sensors desired). A fan and ventilation system were also added to cool the Gumstix**^®^**. Furthermore, the Mk3 buoy had three smaller 5 Watt solar panels connected in parallel. An enhanced more compact telemetry housing was fabricated to neatly contain the hardware.

There were significant enhancements made to the power management system of the Mk3 buoys. A power management circuit was designed to work with the Gumstix**^®^** Overo Air COM allowing the system to be completely suspended between duty cycles. An on-board clock would then wake all of the components when it was time to power up. Furthermore, the end-user was alerted when a buoy was powered up. This enabled a user to connect to the buoy to undertake software maintenance tasks. The power management system kept the buoy powered up until the user disconnects.

As each buoy contained significant on-board computing power, this enabled the implementation of a dynamic duty cycle and power budget. At the beginning of every day the buoy inspects its battery voltage level. The buoy then determines how many times it can power up for the day based on the available power. If there is a series of overcast days whereby the solar panels are unable to sufficiently charge the battery, then the buoy's ‘uptime’ will be reduced to compensate. In the event of a prolonged period of undercharging, the system will go to a “low voltage disconnect” mode and remain down until conditions improve for solar power recharging. The low voltage disconnect (part of the power regulator) prevents the battery from being damaged by overdrawing it when the voltage is too low.

### 5.2. Communications

The same communications setup as the Mk2 was used as it was deemed to be robust and sufficient for the range required at Heron Island (*i.e.*, a 4.5 dBi Superpass antenna on the buoy and a 16 dBi waveguide antenna on the base station communicating via Wi-Fi). However, the major enhancement for the Mk3 was the increased antenna height on the buoy. This time the antenna was positioned at 1.5 m above the water's surface. Note that there was no 3G signal on Heron Island (as is the case on many offshore areas along the Great Barrier Reef), therefore the base station was a requirement to provide Internet access via the Research Station's network infrastructure (*i.e.*, rather than having 3G devices directly on the buoys themselves).

### 5.3. Base Station

**Figure 21.**
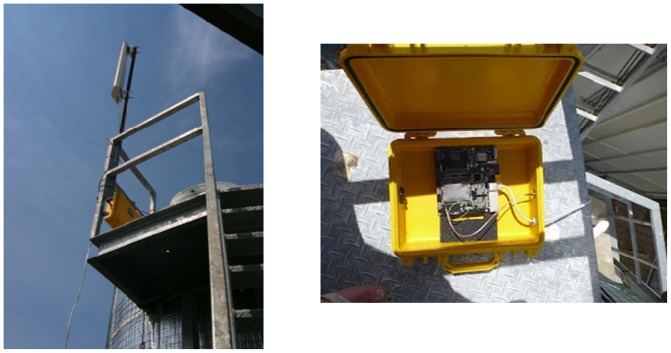
The SEMAT Mk3 base station at the UQ Research Station on Heron Island.

A new base station was developed for use with the Mk3 system. Figure 21 shows the erected base station during the Heron Island deployment. It was fastened on top of a rainwater tank tower that was higher than the surrounding rooves. Again an “off-the-shelf” commodity based approach was used to select the base station hardware (RouterStation Pro**^®^**). The complete base station including the antenna cost approximately $400. This time, the deployment had the luxury of a wired power and Internet connection. Therefore a solar panel, battery and 3G modem were not required.

### 5.4. Sensors

**Figure 22.**
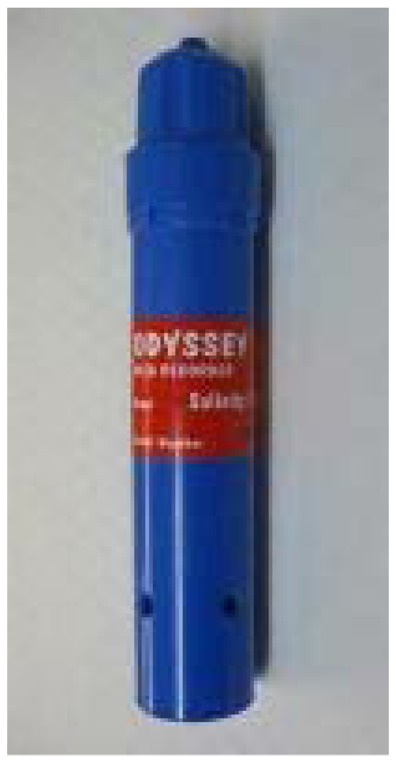
The Odyssey^®^ salinity logger.

The Mk3 introduced a new Odyssey**^®^** sensor for logging salinity levels (see Figure 22). As the sensors and the underwater cabling were the weakest issue with the Mk2 buoys, the design was rigorously scrutinised and a new marine-hardened version was developed. The serial cable was replaced by Versolex**^®^** (www.olex.com.au) underwater power cable. Versolex**^®^** cables are 25 mm in diameter and are commonly used for underwater pumps. The cables have a thick outer insulation housing four wax coated stranded wires. The Odyssey**^®^** logger caps were discarded in favour of a 1 inch BSP threaded to 19 mm barbed director (commonly used for irrigation). The threaded end screwed into the logger (utilising the existing O-ring) and the barbed end fitted on to a Marine Flex**^®^** (www.hosesuppliers.com.au) hose (which contained a spiral helix). The Marine Flex**^®^** hose acted as a flexible outer casing to protect the Versolex**^®^** cable and vent tube from damage (such as fish bites or rubbing against coral). Furthermore, the hose provided an extra layer of waterproofing. A combination of epoxy putty and Araldite**^®^** (a two-part liquid epoxy; www.araldite.com) were used to secure the cable to the director and hose, and also provided protection against water ingress into the logger. The new design was tested successfully up to a depth of 12 m.

### 5.5. Quality Assurance, Error Alerting and Presentation

**Figure 23.**
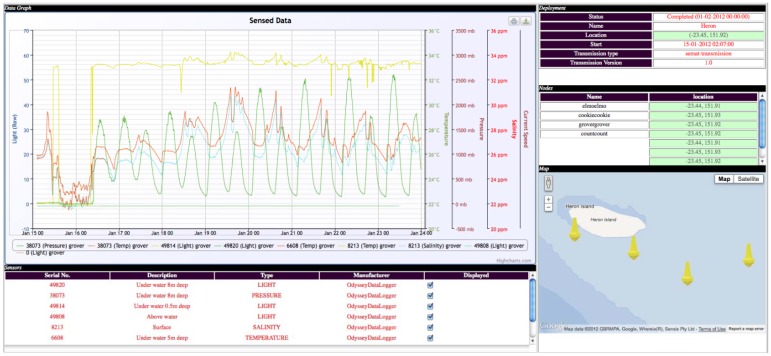
The Mk3 user interface.

A new more sophisticated user interface was developed for the Mk3 deployment (see Figure 23). This interface contained an interactive map (using Google Maps**^®^**) with the buoy locations, error alerting and reporting information, remote power monitoring, data graphing/visualisation tools and quality assurance functionality.

Alert notifications are sent via Google Chat**^®^** so that anyone subscribed to the service could receive status updates via their mobile phone. Ongoing development for the interface is to create a generic software package that can be used by any WSN deployed (SEMAT or otherwise). Towards this goal, the interface successfully consumed data from several AIMS buoys that are located at Heron Island as part of IMOS. A novel feature for this part of the project is the quality assurance techniques. JCU and Griffith University are developing a method based on Fourier series interpolation in an attempt to approximate missing values from the data set. Part of the research involves determining a tolerance factor for how much data can be missing before the accuracy of the interpolated value becomes unreliable.

### 5.6. Data Management—Towards a Standardised Sensor System

The Mk3 incorporated *Sensor Web Enablement* (SWE) standards, which define service interfaces that enable interoperable usage of sensor resources by enabling their discovery, access, tasking, as well as events and alerting [7]. The system model applies the following SWE standards:

- *Observation and Measurements* (O&M)—Standard models and XML schema for encoding observations and measurements from a sensor, both archived and real-time;
- *Sensor Model Language* (SensorML)—Standard models and XML schema for describing sensors, systems and processes associated with sensor observations;
- *Sensor Observation Service* (SOS)—Standard web service interface for requesting, filtering, and retrieving observations and sensor system information;
- *Sensor Alert Service* (SAS)—Standard web service interface for publishing and subscribing to alerts from sensors; and
- *Sensor Planning Service* (SPS)—Standard web service interface for requesting user-driven acquisitions and observations.

**Figure 24.**
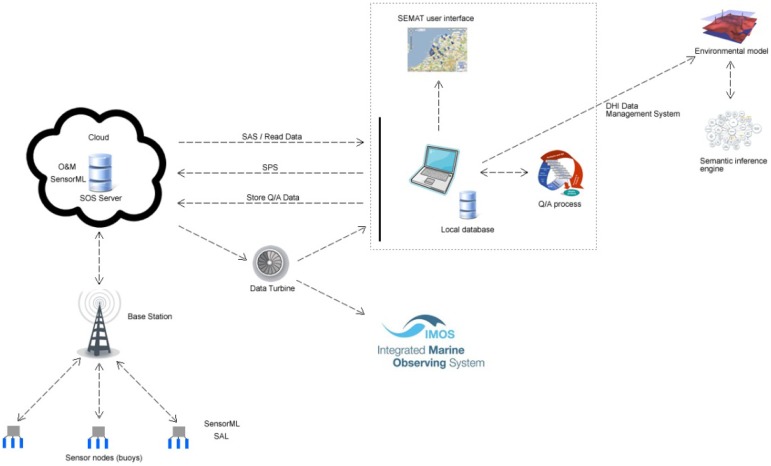
The complete SEMAT Mk3 software architecture.

Figure 24 shows the software architecture as it was implemented for the Mk3 system. SAL interacts with the SOS server using SensorML. SAL focuses on hardware operability, where it manages heterogeneous sensors, and generates the SensorML. The SensorML is transferred to the SOS server for registering the sensors and used as an input file for SAL. The SOS server provides sensor and sensed data information using the SWE standards: SensorML, O&M, SAS and SPS. This allows the SEMAT sensor network management system and other SWE-based applications to control and view the sensed data. The backend of the architecture shows how the WSN integrates with the environmental model and semantic inference engine.

### 5.7. Towards Integrating Semantic Technologies

The Semantic Reef project [23] is an eco-informatics application developed by JCU as an automated data processing, problem-solving and knowledge discovery system to better understand and manage reef ecosystems. The opportunity was taken to establish a linkage between the Semantic Reef and SEMAT, which made possible an end-to-end data collection, analysis and presentation infrastructure.

The data adaptation layer of the SEMAT hierarchy is a bridgeable semantic portal for linked data access, retrieval, synthesis and integration. At this layer the data are semantically enabled through automated annotation via the *Semantic Sensor Web* (SSW) [23] emerging standards. The outcome of this annotation process prepares the data to be a resource available for mapping to the ontologies within the Semantic Reef *Knowledge Base* (KB). Ontologies describe “things” that exist within a domain, whether they are abstract or specific (e.g., a taxonomy of species, anthropogenic influences, a location, a time, *etc.*) to give context and meaning to the data available to the computer [24]. Once the KB ontologies have been populated, the data can be reasoned over and inferences can be made. For example, a domain expert, either a marine scientist of reef manager, can query the KB to extract information of interest, pose observational hypotheses or use as an alert system by inferring events.

The initial coupling of a KB with live data from the SEMAT project established a proof-of-concept focused on the marine science use case. Specifically, marine researchers were able to pose observational hypotheses over a rich range of environmental and anthropogenic factors in real-time from the coupling of these two developing initiatives. Moreover, the infrastructure of SEMAT, once trialed, will be adaptable to many other research disciplines. This flexibility is made possible through the hierarchy of modular ontologies within the KB that range from generic ontologies to domain-specific and application ontologies. The definition of domain-specific ontologies within the KB is all that is required to adapt to an alternative domain such as terrestrial ecology. For example, an ontology to describe the concept of a benthic system could easily be added, through importation, to the Semantic Reef KB.

### 5.8. Towards Underwater Plug and Play of Sensor Technologies and Wireless Power Transfer

UQ (ITEE) investigated the use of the IEEE 1451 standard to enable hardware plug-and-play functionality in the sensor network. The IEEE 1451 standard [25] defines a set of network-independent communication interfaces for connecting transducers (sensors or actuators) to microprocessors. One of the key elements of these standards is the Transducer Electronic Data Sheet (TEDS). TEDS is a memory device attached to the transducer, which stores transducer identification, calibration, correction data, and manufacturer-related information. In particular, the goal of the IEEE 1451 family of standards is to allow the access of TEDS through a common set of interfaces while the transducers are connected to systems or networks via a wired or wireless means, and thus enabling the automatic configuration of the transducer to the system.

A number of projects have been conducted in the past where researchers have attempted to use IEEE 1451 within wireless sensor networks [26]. However, the work in [26] lacks guidelines explaining how similar systems can be developed on different platforms. Furthermore, they use TelosB sensor nodes, which are quite expensive. Previous work by UQ (ITEE) used Arduino technology [21] to model a low-cost prototyping platform, and for this part of the project we demonstrated within a wired network how a TEDS for underwater sensors can be accessed from a low-cost Arduino platform (see Section 4.8).

Our investigations showed that TEDS-enabled sensors still have many challenges. Use of TEDS significantly increases sensor complexity, and there is currently limited software support for TEDS-enabled sensors. This means that significant additional work is required at the central hub to interpret TEDS information. Also TEDS provides standalone fixed data, and does not adequately take advantage of modern networking capabilities. For example, if there are 100 temperature sensors from same manufacturer deployed in a network, it is not necessary to install the full TEDS in all the corresponding slave hubs, since a lot of information is common to all the sensors. If this common information can be sourced from an Internet repository, stored in the central hub and only information unique to sensors (such as individual calibration information) is stored in the corresponding sensors, this would lead to more feasible plug-and-play systems. Note that this part of the project was not used in the Heron Island deployment.

**Figure 25.**
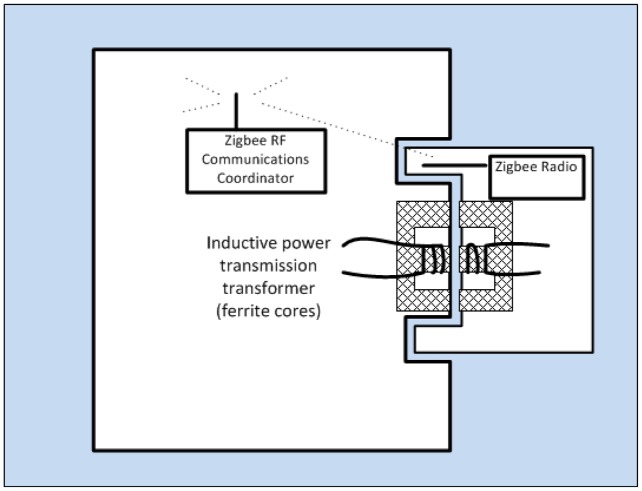
Test setup for underwater wireless transmission.

Another key problem with underwater sensor networks is the replacement or servicing of sensors to maintain system operation in the face of bio fouling, sensor recalibration or saturation, and faulty or damaged sensors. While it is possible to design waterproof connections between sensors and sensor hubs, these require the sensor and the hub to be brought to the surface and dried before the connection can be opened, and a replacement sensor attached. One solution, which allows sensors to be detached and reattached underwater, is to use a wireless connection between the sensor and the hub for both data transmission and power transmission. Our system uses hub and sensor connector housings in immediate proximity to each other, but with no direct electrical connection. For data transmission, conventional wireless radio technology can be used. Experiments with 2.4 GHz Zigbee communications between isolated underwater hubs has shown reliable transmission through approximately 150 mm of fresh water at low power levels (less than 2 mW), and approximately 50 mm in seawater (see Figure 25). Since the antennas will have at most a few mm of water between them, this provides reliable data communications. For power transmission, our design uses a DC-DC converter design, with a two-part ferrite transformer linking the oscillator (hub) and rectifier (sensor) halves of the converter. To ensure good magnetic coupling, our connector design needs to ensure good positional alignment of the two transformer cores. Bench top experiments have shown good power transfer efficiency of 70% or more at low power levels. Progress to date is very encouraging, and we expect that within months will be able to demonstrate a working coupling, which can be detached and re-attached underwater.

### 5.9. Reflections on the Mk3 Development and Deployment

The SEMAT Mk3 buoy was a significant step towards realising SEMAT's goals and was a significant improvement on the Mk1 and Mk2 designs. Some reflections on the Mk3 design post deployment include:

- *There is only so much that can be done to protect a device in a marine environment*. Eventually one must concede boundaries on operating conditions and scale back design and engineering to the costs appropriate to the duration of the deployment. During the Heron Island deployment a severe weather system occurred, with 30–50 knot winds. One of the buoys was ripped from its mooring. The antenna mount was broken, the ballast bar had caused damage to the underside of the buoy, and the benthic sensors had been destroyed (at the Odyssey**^®^** logger's casing level, but not the cables this time).
- *While a test deployment at Magnetic Island off Townsville in North Queensland Australia had proven successful, there was a last minute issue with buoy weight upon the first Mk3 buoy being deployed at Heron Island*. This was largely due to the inclusion of the hatch and repositioning of the solar panels, which was a design that was different to the Magnetic Island test deployment. To rectify the problem, the Mk3 buoys were fitted with a tyre tube to improve stability and floatation. Post deployment analysis and testing showed that the situation could be further improved by using an actual tyre and with enhancements to the ballast bar.
- *The logger failure rate improved from 86% (in the Deception Bay deployment) to 30%*. The majority of destroyed loggers were attributable to the aforementioned buoy that was damaged.
- *There was no integration of the underwater wireless communication and power transfer technologies for the deployment*. This remains a priority challenge for future development of SEMAT.
- *There was limited use of the buoy's full computational power beyond the adaptive duty cycling algorithms*. However, the system's increased capacity now paves the way towards creating a ‘smart’ WSN that has the ability to reason over the data out in the field (*i.e.*, semantic inference).

## 6. Financial Comparison and Discussion

This section contrasts the capabilities of the SEMAT Mk2 and Mk3 prototype buoys. A cost analysis is presented to illustrate the merit of the commodity-based approach. The Mk1 buoy is not analysed as it served as an initial proof-of-concept for securing the NIRAP grant and was narrow in its scope towards realising SEMAT's goals. Table 1 shows the technical specifications for the Mk2 and Mk3 buoys. It can be seen that the Mk3 has the most significant capabilities.

**Figure 26.**
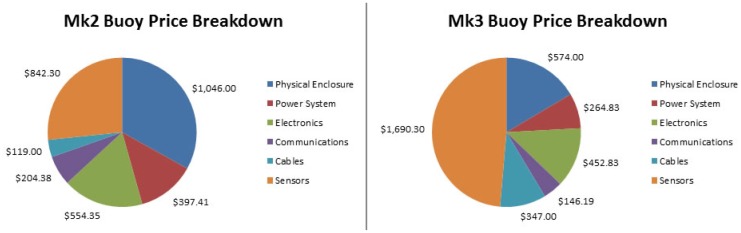
Cost breakdown and comparison for the SEMAT Mk2 and Mk3 buoys.

Figure 26 presents a cost breakdown for Mk2 and Mk3 buoys respectively. Note that the Mk2 is listed at 2010 prices, whereas the Mk3 reflects 2011 prices. The costs are grouped according to whether they are part of the physical enclosure, power system, electronics, communications, cables or sensors. The base station expenses are not included.

The cost does not take into account the research and development effort or the logistical costs of deployment. However, unlike a commercial venture, development outlays do not need to be recovered. This illustrates the importance and merit of using SEMAT's approach to research and development using a combination of universities and industry partners to distribute expenses by employing resources that are part of ordinary work-related duties. The final price for the Mk3 buoy was approximately $4,000 (includes $500 extra for unforeseen expenses that were not accounted for).

It is interesting to note that the sensor cost accounted for 27% of the total for the Mk2 version and 48% of the Mk3. Therefore, the overall costs for the basic architecture without sensors could be as little as $2,000. These costs are the actual expenses associated with instrument packages developed for the research project. As one off, custom units they represent a considerable cost saving compared to the more common IMOS-level solution (discussed in Section 2.2). The costs are set to decrease with underwater communications, development of cheap sensors using the one-wire protocol and future miniaturisation of hardware through applied industrial design and manufacturing.

## 7. Conclusions and Future Directions

This paper described how the SEMAT system has evolved over three years as a proof-of-concept towards creating cost effective marine and coastal environmental monitoring systems. Each SEMAT deployment has been driven by a scientific need to undertake environmental monitoring at a particular site. SEMAT is designed for short-term deployments that are in near coastal, shallow water with relatively calm conditions and is at a pre-commercial stage. While there is some way to go in having a demonstrable high level of reliability in data delivery, this approach has shown how industry can work closely with research institutions to develop better less expensive systems rather than using “one size fits all” solutions to environmental monitoring. Consistent with our original goals for SEMAT we have been able to demonstrate:

1. The ability to adapt and evolve using a commodity-based approach for selecting hardware and software and the benefits of acknowledging Moore's Law to enhance the capabilities WSN's over time.
2. That underwater wireless communications as a feasible, and highly desirable, alternative to cabled systems, particularly in shallow and relative high-energy environments. Our research has also shown that such systems do not appear to pose an environmental threat to electromagnetic sensitive species such as rays and other elasmobranchs.
3. Short-range wireless power transmission issues do remain, as submerged self-powered systems will continue to be dependent on surface-based battery replenishment systems and therefore short-term deployments only. In the SEMAT project considerable effort was focused on the issue of power management with some success (in particular adaptive duty cycling techniques and near total suspension of the electronics when powered down), but this is an area of research that remains a priority.
4. Data can be carried across from a near to real-time measurement system into a management domain such as the Semantic Reef Knowledge Base as well as be used as input to ecological models intended to address environmental management problems. Note that while SEMAT focused on the marine environment, the approach has numerous applications for any other system involving sensors.
5. The goal of minimal deployment expertise remains, but it is anticipated that as SEMAT moves out of the research feasibility and development phase into its next phase of developing country deployments this will be more thoroughly addressed. It was notable that over the period of the last two deployments a very steep learning curve was apparent by the IT specialists and the associated electronics engineering team members as to how demanding the near shore coastal environment can be. This has served as a valuable apprenticeship that has not only improved communications across the research groups but also set us in good stead to deal with the challenges of improving system reliability in an “Expert Free” zone.
6. Our goal of developing a complete package so that the end-user can choose what sensors they require and SEMAT will auto-configure the necessary parameters has been clearly demonstrated as has the ability for the user to be able use a laptop, tablet or smart phone anywhere and begin to view the sensed data with minimal post processing. We are still working on data quality assurance as being an integral part of this package as well as a real-time system status alerting mechanism.

Some avenues for future research and development of the SEMAT system include the following:

- Extending the range and depth that a deployment can be undertaken at—whilst keeping the expense low. To achieve this primary goal is the need for integrating the underwater wireless technologies with the surface buoy.
- Improve upon technologies for the scalability of software and storage and the integration of semantic reasoning. This is of particular importance as sensor networks become more pervasive and the amount of data collected dramatically increases.
- Innovation in energy generation and usage remains an ongoing challenge and this will largely dictate the physical size of the equipment and its flexibility for deployment.
- Incorporating video sensors will open up multiple opportunities in image processing and analysis.
- Interconnecting the SEMAT system with cloud-based storage and computing infrastructure. This is based on the idea of using a distributed database (such as the Hadoop Distributed File System) to stored sensed data and the MapReduce programming model for large-scale parallel data processing.
- The development of a real-time tracking system that dynamically allocates sensor network resources based on which sensors are closest to a phenomenon of interest.

SEMAT is currently being expanded for use as part of the Daintree Rainforest Observatory in far North Queensland Australia. There are also pilot projects underway to monitor water quality in Vietnam's Mekong Delta and around resort areas in Fiji.

## Figures and Tables

**Table 1.** Technical specifications for the Mk2 and Mk3 SEMAT buoys.

	**Mk2 Buoy**	**Mk3 Buoy**
**Computer Hardware**		
Gumstix**^®^**	Overo Air COM**^®^**	Overo Air COM**^®^**
Processor Speed	600 MHz	700 MHz
RAM	256 MB	512 MB
Peripheral Board	Chestnut**^®^**	Summit**^®^**
Fan (Ventilation System)	No	Yes
Number of Serial Hubs	1	2
Battery	12 Volt 7 Amp hours	12 Volt 3.2 Amp hours
Number of Solar Panels	1	3
Solar Panel Specifications	10 Watts	5 Watts
Duty Cycle Period	2 h during the day	Up to 30 min during the day (adaptive based on battery charge trends)
Suspended Power Usage	1 Watt	0.1 Watts
Peak Power Usage	5.5 Watts	6.26 Watts
Low Voltage Disconnect	Yes	Yes
Remote Power Monitoring	No	Yes
**Sensors**		

Temperature	Yes	Yes
Light (PAR)	Yes	Yes
Pressure	Yes	Yes
Salinity	No	Yes
Number of Data Streams	4	8
Logger Failure Rate	86%	32%
**Physical Specifications**		

Telemetry Accessible in the Field	No	Yes
Underwater Sensor Platform	No	Yes
Max Reliable Deployment Duration	2–3 weeks	3–5 weeks
Max Wind Gusts Resilient To	10 knots	40 knots
Reinforced Sensor Cables	No	Yes
Hot-Swapping of Equipment	No	Yes (sensors, power, solar light and telemetry)
Approximate Cost	$3,200	$3,500
